# Pregnancy outcomes in patients with polycystic ovary syndrome who conceived after single thawed blastocyst transfer: a propensity score-matched study

**DOI:** 10.1186/s12884-022-05011-4

**Published:** 2022-09-20

**Authors:** Hui-Ying Jie, Xiu Zhou, Ming-Peng Zhao, Min Hu, Qing-Yun Mai, Can-Quan Zhou

**Affiliations:** 1 Reproductive Medicine Center, The First Affiliated Hospital, Sun Yat-Sen University, Guangzhou, 510080 China; 2 Department of Reproductive Medicine, Department of Obstetrics and Gynaecology, Guangdong Provincial People’s Hospital, Guangdong Academy of Medical Sciences, Guangzhou, China

**Keywords:** PCOS, Blastocyst transfer, Pregnancy, Miscarriage

## Abstract

**Background:**

It remains unclear whether polycystic ovary syndrome (PCOS) is an independent risk factor for pregnancy complications in women undergoing assisted reproductive technology (ART) treatment. For the integrative treatment of PCOS patients, it is still important to investigate the pregnancy outcomes of PCOS patients after adjusting for potential biases, such as body mass index, embryo quality and endometrial preparation method.

**Methods:**

This retrospective cohort study ultimately included a total of 336 PCOS patients who conceived after single thawed blastocyst transfer in the PCOS group and 2,325 patients in the control group from January 2018 to December 2020. A propensity score matching (PSM) model was used, and 336 PCOS patients were matched with 336 patients in the control group.

**Results:**

Before PSM, no differences in the miscarriage rate, pregnancy complication rate, preterm birth rate, or live birth rate were found between the PCOS group and the control group. After PSM, the late miscarriage rate of the PCOS group was significantly higher than that of the control group (3.3% vs. 0.6%, *P* = 0.040), although the early miscarriage rates were similar (14.0% vs. 13.7%). The rates of pregnancy complications, preterm birth and live birth in the PCOS group were comparable to those in the matched control group (*P* = 0.080, *P* = 0.105, *P* = 0.109, respectively). The neonatal weights of male infants and female infants were similar between the two groups (*P* = 0.219, *P* = 0.169). Subgroup analysis showed that PCOS patients with homeostasis model assessment of insulin resistance (HOMA-IR) levels ≥ 2.49 had a significantly increased risk of preterm birth compared with those with HOMA-IR levels < 1.26 and 1.26 ≤ HOMA-IR levels < 2.49 (26.0% vs. 6.0% vs. 9.8%, *P* = 0.005). PCOS patients with total testosterone levels ≥ 0.7 ng/ml had a higher early miscarriage rate but a lower late miscarriage rate than those with total testosterone levels < 0.7 ng/ml (29.4% vs. 12.3%, 0% vs. 3.6%, respectively, *P* = 0.032).

**Conclusions:**

PCOS is an independent risk factor for late miscarriage in patients conceived after a single thawed blastocyst transfer, even after adjusting for biases. Among PCOS patients, insulin resistance and hyperandrogenism are associated with a higher risk of preterm birth and early miscarriage, respectively.

**Supplementary Information:**

The online version contains supplementary material available at 10.1186/s12884-022-05011-4.

## Background

Polycystic ovary syndrome (PCOS) is a prevalent endocrine and metabolic disease in women. According to different definitions, the prevalence rates of PCOS range from 5 to 20% [1]. The characterizing features of women with PCOS usually include clinical or biochemical hyperandrogenism, ovulatory dysfunction, and polycystic ovarian morphology. Women of reproductive age with PCOS usually suffer from obesity and infertility [2, 3]. Assisted reproductive technology (ART) treatment is recommended for patients with PCOS who suffer from infertility.

A growing body of evidence demonstrates that PCOS is associated with pregnancy complications [4, 5]. The risk of premature delivery, gestational diabetes mellitus, and hypertensive disorders of pregnancy in these patients is increased by nearly 2- to 4-fold [5]. High body mass index (BMI) may exacerbate maternal pregnancy complications and neonatal outcomes in PCOS patients [6]. In ART cycles, PCOS patients with high BMI are more prone to suffer from spontaneous abortion than those with normal weight [7]. However, controversies still exist. It has been pointed out that PCOS increases pregnancy complications independent of BMI [8]. In addition, in research investigating the pregnancy outcomes of PCOS patients undergoing IVF, the impact of embryo quality was not considered, which may increase the heterogeneity [9]. Embryo morphology parameters are associated with the pregnancy outcomes of patients undergoing blastocyst transfer [10]. For example, studies have suggested that trophectoderm (TE) quality has an impact on the live birth rate and birth weight [11, 12]. In addition, some studies have suggested that endometrial preparation methods affect pregnancy outcomes, albeit controversy still exists [13–15].

To better assess and treat pregnant PCOS patients, more research is needed to investigate the pregnancy outcomes of PCOS patients after adjusting for potential biases. Therefore, to rule out the potential effect of biases, this study aims to analyze the risk of adverse pregnancy outcomes among PCOS women who conceived after single thawed blastocyst transfer, by applying a propensity score matching (PSM) model.

## Methods

### Study design and subject screening

This retrospective cohort study was conducted in the First Affiliated Hospital, Sun Yat-sen University. From January 2018 to December 2020, PCOS patients first diagnosed with clinical pregnancy after a single thawed blastocyst transfer were initially enrolled (*n* = 431). The inclusion criteria were as follows: i) patients who had a diagnosis of PCOS according to the Rotterdam criteria [16]; ii) patients who underwent IVF/ICSI cycles; and iii) patients for whom a single frozen blastocyst was transferred. Patients who underwent IVF/ICSI due to fallopian tubal factor or male factor infertility were initially enrolled in the control group (*n* = 3267). The exclusion criteria of both the PCOS group and the control group were as follows: 1) preimplantation genetic diagnosis/screening cycles; 2) history of recurrent miscarriage; 3) endometriosis; 4) luteal phase ovarian stimulation; 5) clomiphene citrate administration; 6) metformin administration; 7) congenital uterine malformations; and 8) intrauterine adhesion. This study ultimately included a total of 336 PCOS patients in the PCOS group and 2,325 patients in the control group.

### ART procedures

Stimulation protocols of patients undergoing IVF/ICSI cycles included the gonadotropin-releasing hormone (GnRH) antagonist protocol or GnRH agonist protocol. Gonadotropin was administered to stimulate multiple follicles development. The dosage ranged from 75 to 300 IU per day. Transvaginal ultrasound and blood tests of FSH, LH, estradiol, and progesterone levels were required every three to four days to evaluate follicular development. When at least one dominant follicle had a diameter ≥ 18 mm, human chorionic gonadotropin (HCG) was administered to induce oocyte maturation. Oocyte retrieval was performed 36 h later. Different fertilization methods were performed according to sperm quality. Blastocysts were scored based on the Gardner grading system [17]. In this study, the blastocyst expansion stage was divided into two types, 1) expansion stage < 4 and 2) expansion stage ≥ 4, including expanded blastocysts with a thinning zona, full blastocysts or expanding blastocysts. The inner cell mass (ICM) and TE quality were graded according to the number of ICM or TE cells. As patients underwent FET cycles, single thawed blastocyst transfer was performed under transabdominal ultrasound guidance after endometrial preparation. Patients were administered luteal phase support until the 10^th^ week of gestation. The endometrial preparation protocols included hormone replacement treatment (HRT) cycles, natural cycles (NCs), letrozole-stimulated cycles and GnRH agonist (GnRH-a) cycles: (1) Hormone replacement treatment (HRT) cycles: on Days 2–5 of the menstrual cycle, patients were administered 2–8 mg of estradiol valerate daily (Progynova, Bayer Schering Pharma AG, Berlin, Germany) if the serum estradiol level was < 50 pg/ml and the endometrial thickness was < 5 mm. Serum estradiol level measurements and transvaginal ultrasound scans were performed every 3 to 5 days. If the serum estradiol level was > 100 pg/ml and the endometrial thickness was > 8 mm, progesterone (20 mg per ampoule; Shanghai General Pharmaceutical Co., LTD., China) was administered to achieve endometrial transformation. Then, embryo transfer was carried out on Day 6 of progesterone administration. (2) Natural cycles (NCs): on Days 10 to 12 of the menstrual cycle, transvaginal ultrasound scans and LH urine tests were carried out every 3 to 4 days to monitor the development of the dominant follicle. If the dominant follicle was ≥ 18 mm, 10,000 IU of HCG was injected to induce ovulation. An embryo was transferred on Day 6 after ovulation. (3) Letrozole (LE) cycles: patients took two tablets of letrozole (letrozole tablet, 2.5 mg, Zhejiang Hisun Pharmaceutical Co., LTD., China) daily for five days starting from the second to the fifth day of menstruation. Then, 75–150 units of human menopausal gonadotropin (HMG, Livzon Pharmaceutical Group Inc., China) were injected daily. Transvaginal ultrasound monitoring and serum LH, estradiol, and progesterone level measurements were performed every 3 to 4 days. When a dominant follicle with a diameter above 18 mm was observed, 10,000 IU of HCG was injected to induce oocyte maturation, and embryo transfer was performed on Day 6 after ovulation. (4) GnRH agonist (GnRH-a) cycles: GnRH agonist (1.0 mg, triptorelin, Feiling Pharmaceutical Co., LTD., Germany) was subcutaneously injected. Fourteen days after injection, serum FSH, LH and estradiol level measurements and transvaginal ultrasound scans were carried out to confirm the pituitary downregulation effect. HRT treatment was started on Days 3 to 5 of withdrawal bleeding, and the details were the same as those for the HRT cycles.

### Outcome measures

The homeostasis model assessment of insulin resistance (HOMA-IR) is calculated as follows: fasting insulin × fasting glucose/22.5 [18]. Fasting insulin, fasting glucose and total testosterone (TT) were tested with chemiluminescence.

Supplemental Table 1 shows the definitions of the main outcomes. Clinical pregnancy was defined as the presence of one gestational sac at 7 weeks of gestation after FET [19]. Early or late miscarriages were defined as pregnancy losses before 12 weeks or at 12–24 weeks of gestation, respectively [20]. Pregnancy complications mainly included preeclampsia (defined as gestational hypertension [blood pressure ≥ 140/90 mmHg] or proteinuria plus organ dysfunction after 20 weeks of gestation) [21], pregnancy-induced hypertension syndrome (defined as hypertension [blood pressure ≥ 140/90 mmHg] after 20 weeks of gestation, but resolving up to 12 weeks postpartum) [22], gestational diabetes mellitus (defined as the occurrence or discovery of abnormal glucose metabolism during pregnancy) [23], placenta previa (referred to the lower placental edge overlapping or within 2 cm of the internal cervical orifice in late pregnancy) [24], premature rupture of membrane (the rupture of membranes prior to delivery) [25], fetal distress (referred to the presence of fetal heart trace, with or without fetal acidosis) [26], oligohydramnios (referred to the maximum vertical depth of the amniotic fluid pool or amniotic fluid < 2 cm) [27], and macrosomia (defined as a birth weight ≥ 4000 g) [28]. Preterm birth was defined as delivery after 28 weeks of gestation but before 37 weeks of gestation [29]. Live birth was defined as cycles with at least one live-born baby [19].

### Statistical analysis

All data were analyzed with SPSS software (version 26.0, Inc., Chicago, USA). Continuous variables with a normal distribution are described as the mean and standard deviation. Nonnormally distributed variables are expressed as medians and interquartile ranges. Student’s t test or the Mann‒Whitney U test was used to compare differences between the two groups. Categorical variables, expressed as frequencies and percentages, were analyzed with the chi-square test or Fisher’s exact test. Odds ratios (ORs) and 95% confidence intervals (CIs) were calculated. *P* < 0.05 was considered statistically significant.

With the propensity score matching (PSM) extension of SPSS, a PSM model was performed to minimize bias and increase comparability between the PCOS group and the control group. Four covariates were selected to calculate the propensity score: female age, TE grade, BMI, and the endometrial preparation method. A caliper value was set as 0.02 to ensure matching accuracy. Patients in the PCOS group were matched 1:1 to patients in the control group using the nearest-neighbor random matching algorithm. The sampling method was set as without replacement, and the random number seed was 1 to 6.

## Results

### Baseline characteristics of the population

 A total of 336 PCOS patients were ultimately enrolled in the PCOS group. In addition, 2,325 patients were included in the control group. After 1:1 matching, 336 PCOS patients were successfully matched with 336 patients in the control group. The baseline characteristics of the PCOS group and the control group before and after PSM are shown in Tables 1 and 2, respectively. Before PSM, patients in the PCOS group were younger (30 vs. 32, *P* < 0.001), had a higher BMI (21.6 vs. 20.8, *P* < 0.001), and had a higher level of basal TT (0.42 vs. 0.29, *P* < 0.001) than those in the control group. In addition, the PCOS group had a lower proportion of transferring a C TE grade blastocyst than the control group (5.1% vs. 9.3%, *P* = 0.021). The distribution of endometrial preparation protocols differed between the PCOS group and the control group (*P* < 0.001). In the PCOS group, 75.6% of the patients underwent HRT cycles, which was 1.75 times higher than that in the control group. In addition, 8.3% of the PCOS patients underwent the NC protocol, whereas 44.0% of the non-PCOS patients underwent the NC protocol. After PSM, no differences in maternal age, BMI or TE quality were found, but the endometrial preparation protocol was still different in the two matched groups (*P* < 0.001).

**Table 1:** The baseline characteristics and clinical outcomes in the PCOS group and the control group before PSM

variables	control group	PCOS group	*P*-value
n	2325	336	
Female age (year)	32, 6^a^	30, 5^b^	< 0.001
BMI (kg/m^2^)	20.8, 3.5	21.6, 3.8	< 0.001
< 24	84.6%^a^	75.6%^b^	
≥ 24	15.4%^a^	24.4%^b^	
Basal FSH (IU/L)	5.28, 1.57^a^	4.83, 1.58^b^	< 0.001
Basal LH (IU/L)	3.16, 1.78^a^	5.75, 5.39^b^	< 0.001
Basal TT (ng/mL)	0.29, 0.13^a^	0.42, 0.22^b^	< 0.001
Endometrial preparation			< 0.001
HRT cycles	43.1% (1001/2325)^a^	75.6% (254/336)^b^	
NCs	44.0% (1024/2325)^a^	8.3% (28/336)^b^	
LE cycles	6.1% (142/2325)^a^	12.2% (41/336)^b^	
GnRH-a cycles	6.8% (158/2325)^a^	3.9% (13/336)^b^	
Blastocyst expansion stage			0.386
expansion stage < 4	6.9%	5.7%	
expansion stage ≥ 4	93.1%	94.3%	
Inner cell mass grade			0.276
A	38.3%	42.5%	
B	58.1%	54.8%	
C	3.6%	2.7%	
Trophectoderm grade			0.021
A	22.4%^a^	25.9%^a^	
B	68.3%^a^	69.0%^a^	
C	9.3%^a^	5.1%^b^	
Miscarriage rate	16.4%	17.3%	0.686
Pregnancy complications rate	24.0%	25.6%	0.512
Preterm birth rate	8.2%	10.7%	0.118
live birth rate	81.1%	79.5%	0.471

Non-normally distributed continuous data is presented with median and interquartile range. Categorical data are presented with percentages. *BMI* Body mass index, *FSH* Follicle-stimulating hormone, *LH* Luteinizing hormone, *TT* Total testosterone, *HRT* Hormone replacement treatment, *NCs* Natural cycles, *LE* Letrozole, *GnRH-a* GnRH agonist. Different letters represent statistically significant differences between the two groups (*P*-value < 0.05)

**Table 2:** The characteristics and pregnancy outcomes in the PCOS group and the control group after PSM

	control group	PCOS group	OR (95% CI)	*P*-value
n	336	336	/	
Female age (year)	30, 7	30, 5	/	0.684
BMI (kg/m^2^)	21.6, 4.1	21.6, 3.8	/	0.928
< 24	75.9%	75.6%		
≥ 24	24.1%	24.4%		
Endometrial preparation			/	< 0.001
HRT cycles	59.5% (200/336)^a^	75.6% (254/336)^b^		
NCs	36.3% (122/336)^a^	8.3% (28/336)^b^		
LE cycles	1.2% (4/336)^a^	12.2% (41/336)^b^		
GnRH-a cycles	3.0% (10/336)^a^	3.9% (13/336)^a^		
Trophectoderm grade			/	0.143
A	32.4%	25.9%		
B	64.0%	69.0%		
C	3.6%	5.1%		
Miscarriage rate			/	0.040
early miscarriage rate	13.7% (46/336)^a^	14.0% (47/336)^a^		
late miscarriage rate	0.6% (2/336)^a^	3.3% (11/336)^b^		
Pregnancy complications rate	19.9%	25.6%	1.38 (0.96–1.99)	0.080
Preterm birth rate	7.1%	10.7%	1.56 (0.91–2.68)	0.105
Live birth rate	84.2%	79.5%	0.73 (0.49–1.08)	0.109
Neonatal weight			/	
male infant	3345, 613	3300, 600		0.219
female infant	3200, 550	3150, 625		0.169

Non-normally distributed continuous data is presented with median and interquartile range. Categorical data are presented with percentages. *BMI* Body mass index, *HRT* Hormone replacement treatment, *NCs* Natural cycles, *LE* Letrozole, *GnRH-a* GnRH agonist, *OR* Odds ratio, *CI* Confidence interval. Different letters represent statistically significant differences between the two groups (*P*-value < 0.05)

### Clinical outcomes before and after PSM

Before PSM, the rates of miscarriage, pregnancy complications, preterm birth and live birth in the PCOS group were similar to those in the control group (shown in Table 1). The pregnancy outcomes after PSM are presented in Table 2. The late miscarriage rate of the PCOS group was significantly higher than that of the control group (3.3% vs. 0.6%, *P* = 0.040), although the early miscarriage rates were similar (14.0% vs. 13.7%). The pregnancy complication rate of the PCOS group was slightly higher than that of the control group, but no significant difference was found (25.6% vs. 19.9%, *P* = 0.080; OR 1.38, 95% CI 0.96–1.99). There were no significant differences in the rates of preterm birth and live birth between the PCOS group and the control group (10.7% vs. 7.1%, *P* = 0.105, OR 1.56, 95% CI 0.91–2.68; 79.5% vs. 84.2%, *P* = 0.109, OR 0.73, 95% CI 0.49–1.08, respectively). The neonatal weights in the PCOS group were similar to those in the control group (female infants: 3150 vs. 3200, *P* = 0.169; male infants: 3300 vs. 3345, *P* = 0.219).

Considering that the endometrial preparation protocol might affect clinical outcomes, the Cochran‒Mantel‒Haenszel test was applied (shown in Supplemental Table 2). The results showed that after adjusting for endometrial preparation protocols, the rates of miscarriage, pregnancy complications, preterm birth and live birth were comparable between the two matched groups (*P* = 0.577, *P* = 0.089, *P* = 0.238, and *P* = 0.228, respectively).

### Subgroup analysis in PCOS patients

We further performed subgroup analysis in the PCOS group according to the levels of HOMA-IR, TT and BMI. Supplemental Table 3 displays the pregnancy outcomes of the PCOS patients with different HOMA-IR levels. A total of 202 of the 336 PCOS patients underwent fasting glucose and fasting insulin tests. The HOMA-IR level was stratified into quartiles (25% quartile: 1.26; 75% quartile: 2.49). Compared with the PCOS patients with HOMA-IR levels < 1.26 and 1.26 ≤ HOMA levels < 2.49, the PCOS patients with HOMA-IR levels ≥ 2.49 had a significantly higher preterm birth rate (26.0% vs. 6.0% vs. 9.8%, *P* = 0.005). However, the rates of miscarriage, pregnancy complications and live birth were comparable (*P* = 0.772, *P* = 0.440, *P* = 0.861, respectively).

Considering that the mean TT level of PCOS patients in other studies was nearly 0.7 ng/ml [30, 31], the cutoff value was set as 0.7 in the TT subgroup analysis. We divided the PCOS patients into two subgroups: the TT level < 0.7 ng/ml subgroup (low TT subgroup) and TT level ≥ 0.7 ng/ml subgroup (high TT subgroup). As presented in Table 3, the high TT subgroup had a significantly higher early miscarriage rate but a lower late miscarriage rate than the low TT subgroup (29.4% vs. 12.3%, 0% vs. 3.6%, *P* = 0.032). Correspondingly, the prevalence of pregnancy complications (14.7% vs. 26.8%, *P* = 0.125; OR 0.47, 95% CI 0.18–1.26), preterm birth (5.9% vs. 11.3%, *P* = 0.337; OR 0.49, 95% CI 0.11–2.15) and live birth (70.6% vs. 80.5%, *P* = 0.177; OR 0.58, 95% CI 0.26–1.29) in the high TT subgroup was comparable to that in the low TT subgroup.

**Table 3:** The pregnancy outcomes in PCOS patients with TT level < 0.7 ng/mL and ≥ 0.7 ng/mL

variables	TT < 0.7 ng/mL	TT ≥ 0.7 ng/mL	OR (95% CI)	*P*-value
n	302	34	/	
Miscarriage rate^a^			/	0.032
early miscarriage rate	12.3% (37/302)^a^	29.4% (10/34)^b^		
late miscarriage rate	3.6% (11/302)^a^	0% (0/34)^b^		
Pregnancy complications rate	26.8%	14.7%	0.47 (0.18–1.26)	0.125
Preterm birth rate^*^	11.3%	5.9%	0.49 (0.11–2.15)	0.337
Live birth rate	80.5%	70.6%	0.58 (0.26–1.29)	0.177

Categorical data are presented with percentages. *TT* Total testosterone, *OR* Odds ratio, *CI* Confidence interval. ^*^ indicates Fisher’s Exact Test is used. Different letters represent statistically significant differences between the two groups (*P*-value < 0.05)

Table 4 shows the association between BMI and pregnancy outcomes in the PCOS group. The BMI level was stratified into quartiles (25% quartile: 20.12; 75% quartile: 23.92). BMI quartiles were not significantly associated with the rates of miscarriage, pregnancy complications, preterm birth or live birth (*P* = 0.702, *P* = 0.410, *P* = 0.326, *P* = 0.595, respectively).

**Table 4:** The clinical outcomes in PCOS patients after stratification by BMI quartiles

Variables	BMI < 20.12 kg/m^2^	20.12 ≤ BMI < 23.92 kg/m^2^	BMI ≥ 23.92 kg/m^2^	*P*-value
n	84	168	84	
Miscarriage rate^*^				0.702
early miscarriage rate	17.90%	11.90%	14.30%	
late miscarriage rate	2.40%	4.20%	2.40%	
Pregnancy complications rate	20.20%	28.00%	26.20%	0.410
Preterm birth rate	11.90%	8.30%	14.30%	0.326
Live birth rate	76.20%	81.50%	78.60%	0.595

Categorical data are presented with percentages. *BMI* Body mass index. ^*^ indicates Fisher’s Exact Test is used. *P* < 0.05 was considered statistically significant

## Discussion

In this propensity-matched retrospective study, we found that PCOS patients who conceived after a single thawed blastocyst transfer had a higher risk of late miscarriage than patients without PCOS. Additionally, IR significantly increased the risk of preterm birth in PCOS patients. PCOS patients with a higher level of TT were at increased risk of early miscarriage, and those with a lower level of TT were prone to suffer from late miscarriage.

Previous studies considered PCOS to be an independent risk factor for miscarriage [32, 33]. Due to the impact of anovulation, obesity, IR and androgen excess, sex-hormone receptors, enzymatic pathways and metabolic pathways change abnormally [34, 35]. Hence, the endometrial function of PCOS patients is impaired, which may compromise their pregnancy outcomes. Indeed, a large cohort study found that the miscarriage rate in PCOS patients was 20% higher than that in patients without PCOS [36]. However, other studies have shown inconsistent conclusions. A retrospective cohort study revealed that PCOS patients aged 35 years or older had a higher cumulative pregnancy rate and live birth rate but a miscarriage rate similar to that of age- and BMI-matched control patients [37]. Another study concluded that PCOS patients who conceived through IVF had a similar clinical miscarriage rate compared with patients without PCOS [38]. In contrast, our data demonstrated that PCOS patients were prone to late miscarriage compared with the matched control group. Our results indicated that PCOS was a risk factor for late miscarriage in patients who conceived after FET after adjusting for the biases of age, BMI and embryo quality. Consistent with our results, Cai et al. [32] applied multivariable analysis and found that PCOS increased the risk of late miscarriage, albeit no significant difference was found. They adjusted several important factors, such as age, embryo transfer number and BMI, but did not adjust for embryo quality.

The distribution of endometrial preparation methods differed between the PCOS group and the control group, even after PSM. We found that 75.6% of the PCOS patients underwent HRT cycles, which may be attributed to the oligoovulation and/or anovulation characteristics of PCOS. With the Cochran‒Mantel‒Haenszel test, we found that after PSM, the preparation protocols did not increase pregnancy complications in the PCOS group or the matched control group. Mackens et al*.* [15] pointed out that there is insufficient evidence to support which endometrial preparation protocol is optimal for FET. However, whether endometrial preparation methods affect reproductive outcomes is still controversial. A randomized clinical trial suggested that the NC protocol was superior to HRT cycles [13]. Moreover, Li et al*.* [14] reported that the NC protocol was associated with a decreased risk of obstetric and perinatal complications compared with HRT ovarian stimulation protocols. More prospective randomized clinical trials are required to determine the optimal endometrial preparation method for patients with PCOS.

Notably, we found that PCOS patients with preconception HOMA-IR levels ≥ 2.49 had a higher risk of preterm birth. A large population-based cohort study found that PCOS increased the risk of preterm birth among mothers with type 2 diabetes or GDM, but HOMA-IR levels were not included in this study [39]. IR is detrimental to the decidualization process and glucose homeostasis of the endometrium [35], which may compromise the pregnancy outcomes of PCOS patients. Future research is needed to further validate whether IR increases the risk of preterm birth in PCOS patients and explore the underlying mechanism.

We further applied subgroup analysis in the PCOS group according to different TT levels. We found that a TT level ≥ 0.7 ng/ml was associated with the occurrence of early miscarriage instead of late miscarriage. Chen et al. [40] reported that PCOS patients undergoing fresh embryo transfer had a higher risk of late pregnancy loss than PCOS patients undergoing frozen embryo transfer. In contrast, our results showed that among PCOS patients undergoing single thawed blastocyst transfer, a TT level ≥ 0.7 ng/ml increased the risk of early miscarriage. The mechanisms of miscarriage in patients with PCOS vary. High androgen levels decrease the expression of homeoboxA 10 and integrin, and inhibit glucose transport and angiogenesis of the endometrium among PCOS patients, which may affect endometrial receptivity and further contribute to the occurrence of miscarriage [34]. A high androgen level is detrimental to oocyte and embryo quality [41, 42]. Therefore, the alleviation of hyperandrogenism may be beneficial to patients with PCOS in the early pregnancy stage, but further evidence is needed. In addition, chromosomal abnormalities might contribute to the occurrence of miscarriage in PCOS patients, but the existing evidence is inconsistent [43–45].

In addition, BMI is considered to be associated with compromised pregnancy outcomes. A retrospective cohort study revealed that PCOS patients with a BMI ≥ 30 kg/m^2^ had reduced rates of implantation and live birth and an increased risk of early miscarriage [46]. Chen et al*.* discovered that overweight/obese PCOS patients had a late abortion rate that was nearly three times that of normal-weight PCOS patients after FET [47]. However, it remains inconclusive whether PCOS confers an increased risk of pregnancy complications independent of high BMI [6, 11, 30]. A population-based study [30] found that women with PCOS had a higher prevalence of adverse pregnancy complications, such as GDM, preeclampsia and gestational hypertension, after controlling for obesity and other confounding factors. Similarly, our data suggested that the PCOS group had an increased risk of late miscarriage compared with the matched group. Furthermore, we stratified the PCOS group into three subgroups according to the 25% quartile and 75% quartile of BMI. Previous studies showed that PCOS patients with high BMI had suboptimal pregnancy outcomes [46, 47]. Inconsistently, we found that the pregnancy outcomes were comparable in PCOS patients across different BMI levels. The discrepancy may be due to the limited size of our study.

This cohort study has certain limitations. First, other studies have suggested that metabolic syndrome exacerbates the probability of pregnancy complications in women with or without PCOS [48–50]. The impact of HOMA-IR levels on pregnancy outcomes of PCOS patients was evaluated in our study. However, whether metabolic syndrome confers the risk of adverse pregnancy outcomes could not be analyzed due to the lack of serum lipid profile and waist circumference data. Further investigating the influence of metabolic factors on the pregnancy outcomes of PCOS patients is important and is worthy of further study in the future. In addition, the phenotypes of PCOS may affect pregnancy outcomes. The oocyte competence of PCOS patients varies in different phenotypes [51]. Furthermore, Palomba et al. [52] found that in PCOS patients, trophoblast invasion and macroscopic placental lesions were different across PCOS phenotypes. However, we were unable to perform stratified analysis based on different phenotypes in our study because the PCOS patients were not subdivided by phenotype. Future studies are warranted to evaluate the associations between PCOS phenotypes and pregnancy complications.

## Conclusion

In conclusion, PCOS was an independent risk factor for late miscarriage after matching for age, BMI, TE grade and endometrium preparation method. PCOS patients with IR have an increased risk of preterm birth. In addition, PCOS patients with hyperandrogenism are prone to early miscarriage. These results suggest that PCOS patients with IR and hyperandrogenism require more intensive monitoring when they are pregnant.

## Supplementary Information

**Additional file 1: Supplemental Table 1.** The definitions of clinical outcomes.

**Additional file 2: Supplemental Table 2.** The pregnancy outcomes in the PCOS group and the matched control group with a Cochran-Mantel-Haenszel test adjusting for endometrial preparation methods.

**Additional file 3: Supplemental Table 3.** The pregnancy outcomes in PCOS patients after stratification by HOMA-IR quartiles.

## Data Availability

The datasets generated and/or analysed during the current study are not publicly available due to the hospital policy. However, the datasets are available from the corresponding author on reasonable request and with the permission of the hospital.
