# Supplementary material for: Pregnancy outcomes in patients with polycystic ovary syndrome who conceived after single thawed blastocyst transfer: a propensity score-matched study
Source: BMC Pregnancy Childbirth. 2022 Sep 20;22:718. doi: 10.1186/s12884-022-05011-4 (PMC9487057; doi:10.1186/s12884-022-05011-4)
Supplement: Supplementary file 1 — Additional file 1: Supplemental Table 1. The definitions of clinical outcomes. [file 12884_2022_5011_MOESM1_ESM.docx]

Supplemental Table 1. The definitions of clinical outcomes

| Clinical pregnancy | Defined as the presence of one gestational sac at 7 weeks of gestation after embryo transfer ^[19]^. |
| --- | --- |
| Early miscarriage | Defined as pregnancy loss before 12 weeks of gestation ^[20]^. |
| Late miscarriage | Defined as pregnancy loss between 12-24 weeks of gestation ^[20]^. |
| Preeclampsia | Defined as gestational hypertension [blood pressure ≥ 140/90 mmHg], proteinuria plus organ dysfunction after 20 weeks of gestation ^[21]^. |
| Pregnancy-induced hypertension syndrome | Defined as hypertension [blood pressure ≥ 140/90 mmHg] after 20 weeks of gestation, but resolving up to 12 weeks postpartum ^[22]^. |
| Gestational diabetes mellitus | Defined as the occurrence or discovery of abnormal glucose metabolism during pregnancy ^[23]^. |
| Placenta previa | Refer to lower placental edge overlapping or within 2 cm of the internal cervical orifice in late pregnancy ^[24]^. |
| Premature rupture of membrane | Defined as the rupture of membranes prior to delivery ^[25]^. |
| Fetal distress | Refer to the presence of fetal heart trace, with or without fetal acidosis ^[26]^. |
| Oligohydramnios | Refer to the maximum vertical depth of the amniotic fluid pool or amniotic fluid < 2 cm ^[27]^. |
| Macrosomia | Defined as birth weight ≥ 4000 g ^[28]^. |
| Preterm birth | Defined as delivery after 28 weeks of gestation but before 37 weeks of gestation ^[29]^. |
| Live birth | Defined as cycles with at least one live-born baby ^[19]^. |
