# Supplementary material for: Pregnancy outcomes in patients with polycystic ovary syndrome who conceived after single thawed blastocyst transfer: a propensity score-matched study
Source: BMC Pregnancy Childbirth. 2022 Sep 20;22:718. doi: 10.1186/s12884-022-05011-4 (PMC9487057; doi:10.1186/s12884-022-05011-4)
Supplement: Supplementary file 2 — Additional file 2: Supplemental Table 2. The pregnancy outcomes in the PCOS group and the matched control group with a Cochran-Mantel-Haenszel test adjusting for endometrial preparation methods. [file 12884_2022_5011_MOESM2_ESM.docx]

Supplemental Table 2. The pregnancy outcomes in the PCOS group and the matched control group with a Cochran-Mantel-Haenszel test adjusting for endometrial preparation methods

| variables | HRT cycles (n = 454) | | NC (n = 150) | | LE cycles (n = 45) | | GnRH-a cycles (n = 23) | | OR | 95% CI | *P*-value |
| --- | --- | --- | --- | --- | --- | --- | --- | --- | --- | --- | --- |
|  | Control group  (n = 200) | PCOS group  (n = 254) | Control group  (n = 122) | PCOS group  (n= 28) | Control group  (n= 4) | PCOS group  (n = 41) | Control group  (n = 10) | PCOS group  (n = 13) |  |  |  |
| Miscarriage rate | 15.5% | 18.1% | 13.1% | 10.7% | 0% | 19.5% | 10.0% | 7.7% | 1.17 | 0.74-1.84 | 0.577 |
| Pregnancy  complication rate | 20.5% | 27.2% | 20.5% | 21.4% | 25.0% | 17.1% | 0.0% | 30.8% | 1.43 | 0.97-2.12 | 0.089 |
| Preterm birth rate | 6.0% | 9.4% | 9.0% | 7.1% | 25.0% | 17.1% | 0.0% | 21.3% | 1.51 | 0.83-2.76 | 0.238 |
| Live birth rate | 83.0% | 78.0% | 85.2% | 85.7% | 100% | 80.5% | 90.0% | 92.3% | 0.75 | 0.49-1.15 | 0.228 |

Categorical data are presented with percentages. OR, odds ratio. CI, confidence interval. *P* < 0.05 was considered statistically significant.
