# Supplementary material for: Pregnancy outcomes in patients with polycystic ovary syndrome who conceived after single thawed blastocyst transfer: a propensity score-matched study
Source: BMC Pregnancy Childbirth. 2022 Sep 20;22:718. doi: 10.1186/s12884-022-05011-4 (PMC9487057; doi:10.1186/s12884-022-05011-4)
Supplement: Supplementary file 3 — Additional file 3: Supplemental Table 3. The pregnancy outcomes in PCOS patients after stratification by HOMA-IR quartiles. [file 12884_2022_5011_MOESM3_ESM.docx]

Supplemental Table 3. The pregnancy outcomes in PCOS patients after stratification by HOMA-IR quartiles

| variables | HOMA-IR < 1.26 | 1.26 ≤ HOMA-IR < 2.49 | HOMA-IR ≥ 2.49 | *P*-value |
| --- | --- | --- | --- | --- |
| n | 50 | 102 | 50 |  |
| Miscarriage rate* |  |  |  | 0.772 |
| early miscarriage rate | 20.0% | 16.7% | 12.0% |  |
| late miscarriage rate | 2.0% | 2.0% | 4.0% |  |
| Pregnancy complications rate | 24.0% | 22.5% | 32.0% | 0.440 |
| Preterm birth rate | 6.0%^a^ | 9.8%^a^ | 26.0%^b^ | 0.005 |
| Live birth rate | 76.0% | 79.4% | 80.0% | 0.861 |

Categorical data are presented with percentages. BMI, Body mass index; HOMA-IR, homeostasis model assessment of insulin resistance. * indicates Fisher’s Exact Test is used. Different letters represent statistically significant differences between the two groups (*P*-value < 0.05).
